# National Temporal Trend Analysis of Infective Endocarditis among Patients Infected with HIV in Spain (1997–2014): A Retrospective Study

**DOI:** 10.3390/jcm8081167

**Published:** 2019-08-04

**Authors:** Maria Fe Muñoz-Moreno, Pablo Ryan, Alejandro Alvaro-Meca, Jorge Valencia, Eduardo Tamayo, Salvador Resino

**Affiliations:** 1 Research Support Unit, University Clinical Hospital, 47003 Valladolid, Spain; 2 Internal Medicine Service, University Hospital Infanta Leonor, Madrid, 28031 Madrid, Spain; 3 Gregorio Marañón Health Research Institute (IiSGM), 28007 Madrid, Spain; 4 School of Medicine, Complutense University of Madrid, 28040 Madrid, Spain; 5 Department of Preventive Medicine and Public Health, Faculty of Health Sciences, Universidad Rey Juan Carlos, Alcorcón, 28922 Madrid, Spain; 6 Harm Reduction Unit “SMASD”, Subdirectorate General for Addictions, 28020 Madrid, Spain; 7 Department of Anesthesiology and Resuscitation, University Clinical Hospital, 47003 Valladolid, Spain; 8 Unit of Viral Infection and Immunity, National Center for Microbiology—Carlos III Health Institute, Majadahonda, 28222 Madrid, Spain

**Keywords:** HIV/AIDS, infective endocarditis, etiology, epidemiology, ICD9CM codes

## Abstract

Background: People living with human immunodeficiency virus (HIV) (PLWH) form a vulnerable population for the onset of infective endocarditis (IE). We aimed to analyze the epidemiological trend of IE, as well as its microbiological characteristics, in PLWH during the combined antiretroviral therapy era in Spain. Methods: We performed a retrospective study (1997–2014) in PLWH with data obtained from the Spanish Minimum Basic Data Set. We selected 1800 hospital admissions with an IE diagnosis, which corresponded to 1439 patients. Results: We found significant downward trends in the periods 1997–1999 and 2008–2014 in the rate of hospital admissions with an IE diagnosis (from 21.8 to 3.8 events per 10,000 patients/year; *p* < 0.001), IE incidence (from 18.2 to 2.9 events per 10,000 patients/year; *p* < 0.001), and IE mortality (from 23.9 to 5.5 deaths per 100,000 patient-years; *p* < 0.001). The most frequent microorganisms involved were staphylococci (50%; 42.7% *Staphylococcus aureus* and 7.3% coagulase-negative staphylococci (CoNS)), followed by streptococci (9.3%), Gram-negative bacilli (8.3%), enterococci (3%), and fungus (1.4%). During the study period, we found a downward trend in the rates of CoNS (*p* < 0.001) and an upward trends in streptococci (*p* = 0.001), Gram-negative bacilli (*p* < 0.001), enterococci (*p* = 0.003), and fungus (*p* < 0.001) related to IE, mainly in 2008–2014. The rate of community-acquired IE showed a significant upward trend (*p* = 0.001), while the rate of health care-associated IE showed a significant downward trend (*p* < 0.001). Conclusions: The rates of hospital admissions, incidence, and mortality related to IE diagnosis in PLWH in Spain decreased from 1997 to 2014, while other changes in clinical characteristics, mode of acquisition, and pathogens occurred over this time.

## 1. Introduction

Infective endocarditis (IE) is uncommon but causes high morbidity and mortality [1]. In recent decades, the epidemiology of IE has evolved in developed countries. The population at risk of infection used to be people with rheumatic valvular heart disease, those with a history of dental interventions or intravenous drug users. However, in recent decades, the incidence of IE has increased in elderly people and patients who undergo in-hospital procedures [1,2]. In Spain, Olmos et al. found that the IE incidence in the general population increased from 2.72 in 2003 to 3.49 per 100,000 person-years in 2014, in a work published with data from the Minimum Basic Data Set (MBDS) from Spain [3]. Several population-based studies in Europe have also showed an increase in IE incidence in the general population [4,5,6,7] and in intensive care units [8] over time. Regarding the responsible infectious agents, streptococci and staphylococci are the most common cause of IE (approximately 80%) in most developed countries, but the proportion of these two microorganisms varies by region and year. Enterococci are the next most common cause of IE and, less commonly, fungi and Gram-negative bacilli [1].

During the history of the human immunodeficiency virus (HIV) epidemic, approximately 75 million people worldwide have been infected with HIV and, currently, there are approximately 37 million people living with HIV (PLWH) [9]. In the absence of combined antiretroviral therapy (cART), HIV-infected subjects have a progressive CD4+ T cell loss and a wide range of immunological abnormalities, which lead to an increased risk of infections [9]. Since the mid-90s, cART has led to durable HIV suppression, which allowed immune recovery and a dramatic reduction in the risk for developing acquired immune deficiency syndrome (AIDS) [9]. However, despite effective cART, the HIV-infected population is ageing prematurely, and the onset of frailty occurs at an earlier age [10]. Thus, HIV-infected patients on cART, even after achieving an acceptable immunological status, still remain a “fragile” population [11] with a higher risk of bloodstream infections and IE [12].

The CD4+ T-cell count is a key factor in relation to the risk of infection in PLWH [13,14], even in HIV-infected patients on cART [15], since a dysregulation of the immune system predisposes an individual to invasive infections and the development of IE [12,13,16]. HIV-infected patients with CD4 cell counts >500 cells/μL had a higher risk of infections compared to those with a CD4 count >750 cells/µL [15]. Thus, the viability of cART and the effectiveness of these antiretroviral treatments may have been a key factor in the epidemiology of IE in the last two decades. There is little knowledge of IE epidemiology in PLWH during the cART era in regards to rates of hospital admission, incidence, and mortality in large cohorts or in population studies.

## 2. Objective

We aimed to analyze the epidemiological trends of IE, as well as the associated microbiological characteristics, in PLWH during the cART era (1997–2014) in Spain by using the Minimum Basic Data Set (MBDS) of the Spanish National Health Service.

## 3. Materials and Methods

### 3.1. Study Population

A nationwide population-based retrospective study was performed in HIV-infected patients with an IE diagnosis between January 1, 1997 and December 31, 2014 in Spain. The study period was stratified into four calendar periods: (a) 1997–1999; (b) 2000–2003; (c) 2004–2007; (d) 2008–2014. All subjects were older than 18 years.

Data were obtained from discharge records in the Spanish MBDS of the Ministry of Health, Consumption and Social Welfare (MHCSW). Data were requested in 2017, but the MHCSW gave us the historical series until 2014 because the Spanish MBDS releases the data with a 2–3-year lag. Then, debugging the acquired database was carried out. The MBDS is an administrative database, which contains epidemiological and clinical information recorded at the time of hospital discharge. The discharge diagnoses and procedures performed during the hospital stay were recorded according to the International Classification of Diseases, 9th ed, Clinical Modification (ICD-9-CM). The MDBS has a coverage of 92% of all Spanish hospitals, 84.14% of which are public hospitals and 15.86% are private hospitals [17]. The Spanish MHCSW ensured the data quality by establishing protocols for record keeping and conducting periodic audits on the MBDS.

**Figure 1:**
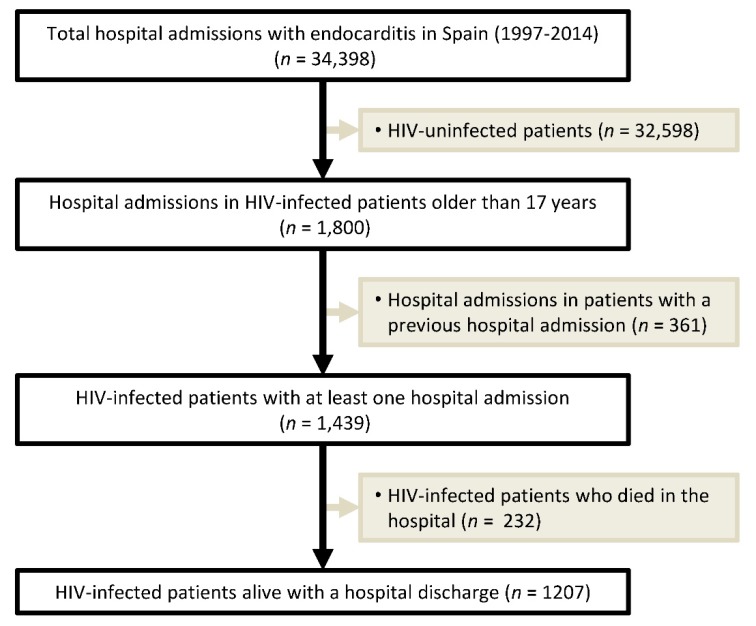
Flow chart of the selection of human immunodeficiency virus (HIV)-infected patients in Spain included in the study (1997 to 2014).

The clinical status of patients was defined by ICD-9-CM codes (see Supplementary Table S1). Overall, 34,398 hospital admissions with an IE diagnosis were found from 1997 to 2014 in the Spanish MBDS. Among those, 1800 were admissions of HIV-infected patients (ICD-9-CM codes: 042 or V08), which corresponded to 1439 patients with at least one hospital admission (Figure 1).

### 3.2. Ethics Statement

The health-related personal data were requested by the MHCSW from Spanish hospitals, according to Spanish legislation [18]. Additionally, the identification of the patient was encrypted and anonymized. Therefore, the signed patient’s consent was not needed because the MBDS information is mandatory and it is an anonymous dataset. The MHCSW and the Research Ethics Committee (Comité de Ética de la Investigación y de Bienestar Animal; CEI PI 69_2012) of the Instituto de Salud Carlos III (Madrid, Spain) approved our study. The signed patient’s consent was not needed because the Minimum Basic Data Set contains mandatory information and it is an anonymous dataset.

### 3.3. Study Variables and Statistical Analysis

The main outcome was the IE diagnosis (ICD-9-CM codes: 421.0, 421.1, and 421.9), which was diagnosed according to standard procedures in each hospital of the Spanish National Health System. In this study, we defined the following outcome variables and rates related to IE: (i) hospital admission with an IE diagnosis (any admission that had a diagnosis of IE included in any position.); (ii) first IE diagnosis or index episode (incidence of new IE diagnosis, patients readmitted with a later IE diagnosis were discarded); (iii) in-hospital death among patients with IE diagnosis (mortality and case fatality rate). The rate of hospital admissions (episodes per 10,000 person-years), incidence (new episodes per 10,000 person-years), and mortality (deaths per 100,000 person-years) were calculated by the ratio between the number of events and the number of persons at risk within each calendar period, according to the number of PLWH in Spain (reference population), which was provided by the National Centre of Epidemiology (Instituto de Salud Carlos III, Madrid, Spain) (see Supplementary Table S2) [19]. The case fatality rate was calculated as the ratio between the number of deaths and the number of patients with an IE diagnosis (percentage, %).

Next, we analyzed the rate of hospital admissions with an IE diagnosis according to mode of acquisition: (i) community-acquired IE (only main diagnosis of IE) and (ii) health care-associated IE (there was no main diagnosis of IE, but there was a secondary diagnosis). We also analyzed the presence of codes for specific microorganisms in the hospital admissions with an IE diagnosis (see Supplementary Table S1): staphylococci (coagulase-negative staphylococci (CoNS) and *Staphylococcus aureus*), streptococci, enterococci, Gram-negative bacilli, and fungus. The rate of each microorganism was calculated as the ratio (percentage) between the number of times the microorganism was found and the number of those cases with a diagnosis of IE.

The chi-squared test and Fisher’s exact test were used to analyze categorical data and proportions, as required. Continuous variables were studied using the *t*-Test or Mann–Whitney *U* test. The Extended Mantel–Haenszel Chi-Square was used to evaluate the temporal trends of rates. All statistical tests were performed with the Statistical Package for the Social Sciences (SPSS) 22.0 software (IBM Corp., Chicago, IL, USA) and were considered significant with values of *p* < 0.05 (two-tailed).

## 4. Results

### 4.1. Characteristics of the Study Population

Table 1 shows the characteristics of 1800 hospital admissions of HIV-infected patients with an IE diagnosis in Spain (1997 to 2014).

Overall, most of the patients were men (83%) with a median age of 36 years. When the characteristics of the patients were stratified by calendar periods, we found a significant increase in the age strata (*p* < 0.001), percentage of patients with substance abuse (drugs (<0.001), alcohol (*p* = 0.024), and tobacco (p < 0.001)), and some comorbidities (diabetes (*p* < 0.001), hypertension (*p* < 0.001), coronary artery disease (*p* = 0.004), liver disease (*p* < 0.001), chronic obstructive pulmonary disease (*p* < 0.001), and chronic kidney disease (*p* < 0.001)). We also found an upward trend in predisposing factors such as congenital heart disease (*p* = 0.008), valve surgery (*p* = 0.029), infection of cardiac device or implant (*p* < 0.001), history of pacemaker or defibrillator placement (*p* = 0.025), history of prosthetic valve replacement (*p* = 0.009), receiving intravenous therapy or home care (*p* < 0.001), and hemodialysis (*p* < 0.001). Viral hepatitis was the most frequent coinfection (43.5%), particularly chronic hepatitis C (40.1%). During the follow up, we found a significant upward trend of hepatitis B (from 4 to 10.3; *p* < 0.001) and chronic hepatitis C (from 14.5 to 60.3; *p* < 0.001). Mycobacteria was another frequent microorganism (2.8%), but we found a significant decrease in mycobacteria infection from 1997–1999 to 2008–2014 (from 3.7 to 2.5; *p* < 0.001). The percentage of patients with one or more than two organ failures was 12.7 and 2.9%, respectively, and increased during the follow up.

### 4.2. Epidemiological Trends of Infective Endocarditis

**Figure 2:**
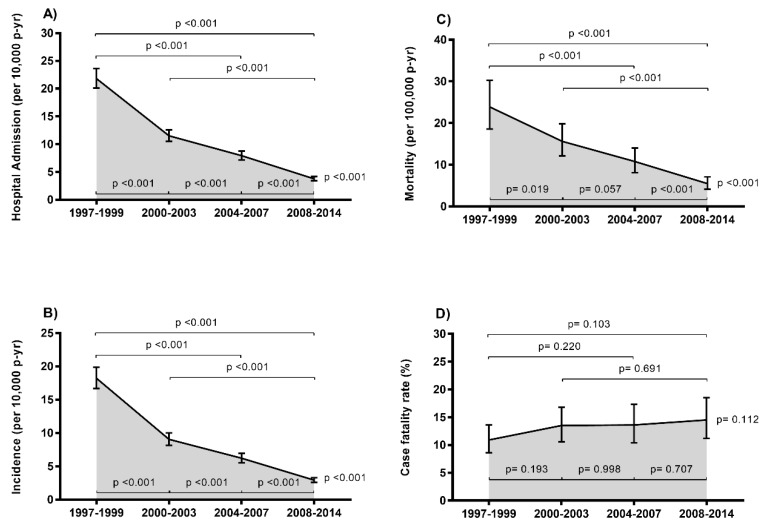
Epidemiological trends of infective endocarditis in HIV-infected patients in Spain (1997 to 2014). (**A**) Hospital admissions. (**B**) Incidence; (**C**) Mortality; (**D**) Case fatality rate. Statistical: Differences between groups were calculated by the Chi Square test. Linear trends from 1997–1999 to 2008–2014 calculated by the Extended Mantel–Haenszel Chi Square.

The trends of the rates of IE in HIV-infected patients in Spain during the period from 1997 to 2014 are shown in Figure 2 (full description in Supplementary Table S3). We found significant downward trends from 1997–1999 to 2008–2014 in the rate of hospital admissions with an IE diagnosis (from 21.8 to 3.8 events per 10,000 patients/year; *p* < 0.001), IE incidence (from 18.2 to 2.9 events per 10,000 patients/year; *p* < 0.001), and IE mortality (from 23.9 to 5.5 deaths per 100,000 patient-years; *p* < 0.001). However, the case fatality rate (approximately 12.9%) did not change during follow up.

### 4.3. Epidemiological Trends of IE-Related Microorganisms

**Figure 3:**
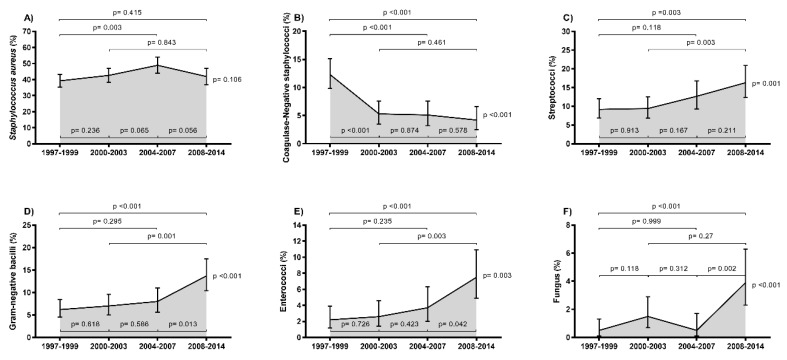
Epidemiological trends of causative microorganisms of infective endocarditis in HIV-infected patients in Spain (1997 to 2014). (**A**) *Staphylococcus aureus*. (**B**) Coagulase-Negative staphylococci. (**C**) Streptococci. (**D**) Gram-negative bacilli. (**E**) Enterococci. (**F**) Fungus. Statistical: Differences between groups were calculated by the Chi Square test. Linear trends from 1997–1999 to 2008–2014 calculated by the Extended Mantel–Haenszel Chi Square.

The trends in the rates of IE by type of microorganism in HIV-infected patients in Spain during the period from 1997 to 2014 is shown in Figure 3 (full description in Supplementary Table S4). 

Overall, the most frequent microorganisms involved were staphylococci (50%; 42.7% *Staphylococcus aureus* and 7.3% CoNS), followed by streptococci (9.3%), Gram-negative bacilli (8.3%), enterococci (3%), and fungus (1.4%). During the study period, the rates for *Staphylococcus aureus* remained consistent without major changes, but we found a significant downward trend in the rate of CoNS (*p* < 0.001), particularly between the first calendar period (1997–1999) and the other calendar periods. Moreover, we found a significant upward trend in the rates of streptococci (*p* = 0.001), Gram-negative bacilli (*p* < 0.001), enterococci (*p* = 0.003), and fungus (*p* < 0.001), mainly in 2008–2014. We also emphasize that only 37 of *S. aureus* isolates were methicillin-resistant *S. aureus* (MRSA) and the MRSA rate (around 2%; 95% CI = 1.5%; 2.7%) did not have significant changes during the follow up.

### 4.4. Epidemiological Trends of IE According to Mode of Acquisition

**Figure 4:**
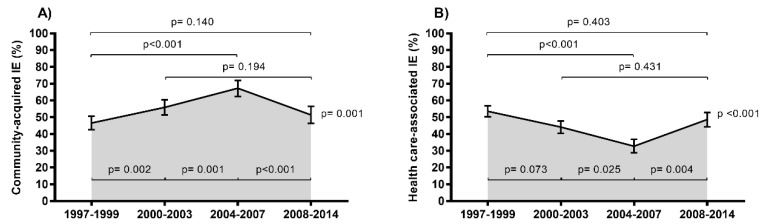
Epidemiological trends of infective endocarditis (IE) in HIV-infected patients in Spain (1997 Table 2014 stratified by modes of acquisition. (**A**) Community-acquired IE. (**B**) Health care-associated IE. Statistical: Differences between groups were calculated by the Chi Square test. Linear trends from 1997–1999 to 2008–2014 calculated by the Extended Mantel–Haenszel Chi Square.

The rates of IE stratified by mode of acquisition (community-acquired vs. health care-associated) are shown in Figure 4 (full description in Supplementary Table S5). 

**Figure 5:**
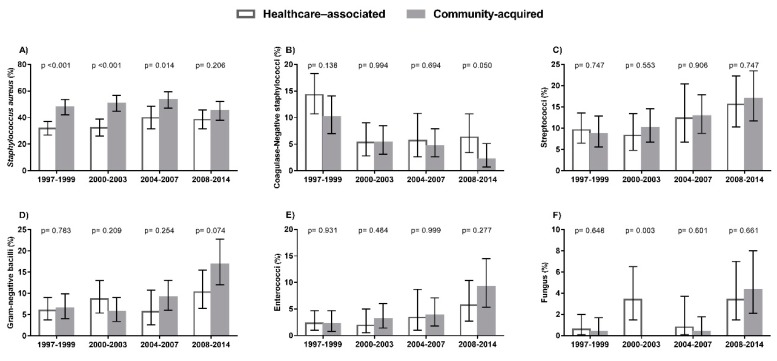
Epidemiological trends of causative microorganisms of infective endocarditis in HIV-infected patients in Spain (1997 to 2014) stratified by health care-associated and community-acquired endocarditis. (**A**) *Staphylococcus aureus*. (**B**) Coagulase-Negative staphylococci. (**C**) Streptococci. (**D**) Gram-negative bacilli. (**E**) Enterococci. (**F**) Fungus. Statistical: Differences between groups were calculated by the Chi Square test. Linear trends from 1997–1999 to 2008–2014 calculated by the Extended Mantel–Haenszel Chi Square.

Overall, the rate of community-acquired IE showed a significant upward trend (from 46.5% to 51.4%; *p* = 0.001), while the rate of health care-associated IE showed a significant downward trend (from 53.5% to 48.6%; *p* < 0.001), with the differences between the two modes of acquisition being most prominent in the 1997–1999 (*p* = 0.016), 2000–2003 (*p* < 0.001), and 2004–2007 (*p* < 0.001) periods. However, we found a significant change in trend between the periods 2004–2007 and 2008–2014, both for community-acquired IE (*p* < 0.001) and health care-associated IE (*p* = 0.004). Furthermore, we also analyzed the rates of IE-related microorganisms stratified by modes of acquisition (Figure 5; full description in Supplementary Table S6). Overall, *Staphylococcus aureus* cases were more frequent in the community-acquired IE (49.5% vs. 34.5%; *p* < 0.001), with these differences being more significant in the 1997–1999 (*p* < 0.001), 2000–2003 (*p* < 0.001), and 2004–2007 (*p* = 0.014) periods.

## 5. Discussion

In this retrospective study on PLWH, we analyzed the epidemiological changes in IE in Spain during a 17-year period, from 1997 to 2014. Overall, we found downward trends in hospital admissions, incidence, and mortality related to IE diagnosis, but we also found significant changes in IE patient characteristics (age, substance abuse, comorbidities, predisposing factors, and number of organ failures), mode of acquisition (community-acquired IE vs. health care-associated IE), etiology (IE-related microorganisms), and coinfections (non-IE-related microorganisms). To our knowledge, our study is the first report evaluating the nationwide epidemiological trends of IE in PLWH during the cART era (1997–2014).

Regarding patient characteristics, we want to highlight that the prevalence of comorbidities, predisposing factors, and number of organ failures higher than two were low in PLWH with a diagnosis of IE; however, we found increases during the follow up in the prevalence of these characteristics and we also found increases in age and substance abuse. We do not know exactly the number of active intravenous drug users (IVDUs) because ICD-9-CM only has diagnosis codes that define substance use disorders [20]. However, epidemiological data available in Spain indicate that the percentage of active IVDUs of all HIV-infected patients has decreased on aggregate [21]. The most frequent coinfection was HCV infection, and an upward trend of hepatitis B and HCV infection during follow up was found. However, it should be noted that the Spanish MBDS only collects hepatitis serology data and, therefore, all patients exposed to HCV and HBV are included here (clarifiers, infected, and cured). The percentage of subjects who resolve HBV and HCV infection spontaneously is high, particularly in hepatitis B. In addition, the percentage of subjects cured of hepatitis C, who achieved sustained virological response after antiviral therapy, has increased over the years. It is most likely that increased life expectancy in HIV-infected patients with chronical viral infections and the emergence of more effective treatments may be responsible for this increase in the prevalence of hepatitis C [22,23] and hepatitis B [24,25]. Another relevant coinfection was mycobacteria, with a downward trend from 1997 to 2014, which is consistent with previously published data on the entire HIV population of Spain [26]. These epidemiological trends may be related to the increasing use of cART during the previous decades in Spain [26], which has improved the immunological status of patients [27] and decreased their risk of infections [28].

In our study, we found a downward trend in the rates of incidence and hospital admissions related to IE in PLWH in Spain. It is very likely that the decrease observed in IE in our study may be due to the widespread use of cART. In developed countries, one of the main factors that impacts the risk for development of bloodstream infections is the availability of cART [29], which promotes HIV suppression, immune recovery, and a lower risk of infections [9,30]. The Spanish National Health System (NHS) provides free medical care to 99.5% of the Spanish population and the rate of HIV-infected individuals on cART increased significantly over the study period [27], increasing the proportion of patients with undetectable HIV viral load and high CD4 T-cells count, and decreasing the number of patients in an advanced clinical stage [27]. Also, during the follow-up period, cART has improved its effectiveness [31], and due to simpler and less toxic drugs, adherence has increased [32], which could have contributed to a decrease in the IE risk. Despite this, our data on IE incidence in Spanish PLWH was 10 times higher than in the Spanish general population in the last calendar period [3]. HIV-infected patients on suppressive cART are still a “fragile” population with an increased risk for acquiring IE [12], since subjects with CD4+ T-cells count > 500 cells/μL continue to be at risk of infection compared to patients with CD4 count > 750 cells/µL [15]. Moreover, Tsabedze et al. reported in a recent article that the prognosis of IE in HIV-infected patients is similar to that for their HIV-negative counterparts, but HIV-positive patients with low CD4 counts still have a higher mortality rate [33]. In our study, the IE mortality decreased during follow up, as the IE incidence decreased. This trend in IE mortality is clearly influenced by a decrease in the number of IE cases. However, the case fatality rate did not change during the study period, and these values were slightly lower than values recently reported in the Spanish general population, with the same database (MBDS), and a similar follow-up period [3]. Therefore, although HIV-infected patients were at higher risk for IE, their mortality was not higher than the general population.

Regarding the microbiological profile of IE episodes, we found that the most frequent microorganisms involved in incident IE were staphylococci, particularly *Staphylococcus aureus*, followed by streptococci, Gram-negative bacilli, enterococci, and fungus. In this study, we could only analyze streptococcal infections without specifying species, such as *Streptococcus viridans*, because this microorganism does not have a specific ICD-9 code. For this reason, we included ICD-9 codes for group A, B, C, and G streptococci, as well as unspecified streptococcal infections. Despite this, we think most of the streptococci were *Streptococcus viridans*, since the microbiological profile of IE supports it [34].

A main factor that influences the epidemiology of IE is the geographic distribution of some pathogens [34]. In this sense, our data are not consistent with a recent report on the Spanish general population [3] or with most of the studies published in Europe [34]. In relation to the Spanish general population, Olmos et al. [3] found in the Spanish MBDS that the rates of *S. aureus* and streptococci had a downward trend from around 20 and 23% at baseline (2003) to approximately 15 and 19% at the end of follow up (2014), respectively. In our study, we found that the rate of *S. aureus* was higher (approximately 43%) and had not changed significantly during the follow up, and the streptococci rate was lower (approximately 10%) and with a more significant upward trend during follow up. In addition to this, Olmos et al. [3] found a significant upward trend in the CoNS rate while we found that the CoNS rate in PLWH remained stable during the same period. Another notable difference was that Olmos et al. [3] found an enterococci rate of approximately 13%, which exceeds 15% at the end of the follow up, double what was found in our data. All these differences indicate that HIV-infected patients and the general population had a different distribution of IE-related microorganisms in Spain.

Staphylococci infections are frequent among HIV-infected active IVDUs with IE, particularly in the community-based setting [34]. In our study, *S. aureus* was by far the most commonly isolated microorganism among community-acquired cases, but this difference with respect to health care-associated cases disappeared in the last calendar period. Additionally, the decline in the CoNS rates found in our study could have been related to the decrease in the rate of active IVDUs among Spanish HIV-infected patients [21]. The rise in the rates of streptococci, Gram-negative bacilli, enterococci, and fungus could be related to the greater use of health care–related interventional procedures in recent years. However, when we performed an analysis of the IE-related microorganisms stratified by health care-associated and community-acquired IE, we did not find significant differences in these four groups of microorganisms.

## 6. Study Limitations

Firstly, our study was retrospective, using an administrative database (MBDS). This entails a series of limitations that could have caused a confusion bias: (i) an absence of relevant information such as treatments, definite and possible IE episode, therapeutic procedures, prognostic scores (Child Pugh Turcotte (CPT), Model for End-stage Liver Disease (MELD), or Acute Physiology and Chronic Health Evaluation (APACHE), or Sequential Organ Failure Assessment (SOFA)), previous hospitalizations, etc.; (ii) we did not have data on the potential accuracy of the Spanish MBDS for IE-related diagnoses, but given the specific features and relevance of IE, incorrect coding should be less likely than for other diseases; (iii) a lack information related to the type of acquisition (nosocomial, community acquired), which was implemented based on the timing of the IE code (main or secondary diagnosis); (iv) there was a lack of microbiological information in 30% of hospital admissions (culture-negative or not recorded), and consequently, the rates may be underestimated; (v) MBDS data are anonymous and it is difficult to identify whether a patient has been hospitalized more than once in different hospitals, but we performed an analysis of patient codes and dates of admission and discharge to detect hospital changes or re-admissions with IE; (vi) no data were available from a significant percentage of private hospitals, so the rates may be underestimated. However, these private centers represent a small percentage of the total centers in Spain.

Secondly, we did not have data on the total number of PLWH in Spain between 1997 and 2014, because there were no data on the national coverage of HIV diagnoses during this period. Instead, we used estimates of the number of PLWH provided by the National Centre of Epidemiology (NCE, Instituto de Salud Carlos III, Madrid, Spain), but without stratifying by gender and age. Therefore, we could not calculate the rates stratified by gender and age.

Thirdly, our data were recorded before 2014 (more than four years ago) and our findings may not reflect the current characteristics and outcomes related to IE.

## 7. Conclusions

Our data show that the rates of hospital admissions, incidence, and mortality related to IE diagnosis in PLWH in Spain decreased from 1997 to 2014, while other changes in clinical and epidemiological characteristics, mode of acquisition, and pathogens occurred over this time.

## Figures and Tables

**Table 1:** Clinical and epidemiological characteristics of hospital admissions of HIV-infected patients with infective endocarditis in Spain (1997 to 2014).

	All Patients	Calendar Periods				
		1997–1999	2000–2003	2004–2007	2008–2014	*p*-Value
Number of patients	1800	594	474	376	358	
Male	1494 (83%)	496 (83.5%)	398 (84.3%)	302 (80.3%)	298 (83.2%)	0.454
Age, years						
<30	413 (22.9%)	251 (42.3%)	86 (18.2%)	45 (12%)	31 (8.7%)	<0.001
30–34	405 (22.5%)	159 (26.8%)	136 (28.8%)	68 (18.1%)	42 (11.7%)	<0.001
35–39	439 (24.4%)	116 (19.5%)	135 (28.6%)	118 (31.4%)	70 (19.6%)	0.301
40–44	302 (16.8%)	50 (8.4%)	72 (15.3%)	101 (26.9%)	79 (22.1%)	<0.001
>45	241 (13.4%)	18 (3%)	43 (9.1%)	44 (11.7%)	136 (38%)	<0.001
Substances of abuse						
Drugs	386 (21.4%)	73 (12.3%)	91 (19.3%)	110 (29.3%)	112 (31.3%)	<0.001
Alcohol	16 (0.9%)	2 (0.3%)	3 (0.6%)	5 (1.3%)	6 (1.7%)	0.003
Tobacco	358 (19.9%)	56 (9.4%)	84 (17.8%)	105 (27.9%)	113 (31.6%)	<0.001
Comorbidities						
Diabetes	25 (1.4%)	1 (0.2%)	7 (1.5%)	5 (1.3%)	12 (3.4%)	<0.001
Hypertension	25 (1.4%)	1 (0.2%)	5 (1.1%)	4 (1.1%)	15 (4.2%)	<0.001
Coronary artery disease	15 (0.8%)	1 (0.2%)	2 (0.4%)	6 (1.6%)	6 (1.7%)	0.004
Peripheral vascular disease	51 (2.8%)	9 (1.5%)	15 (3.2%)	19 (5.1%)	8 (2.2%)	0.148
Cerebrovascular Disease	25 (1.4%)	5 (0.8%)	6 (1.3%)	10 (2.7%)	4 (1.1%)	0.317
Cancer	16 (0.9%)	4 (0.7%)	4 (0.8%)	2 (0.5%)	6 (1.7%)	0.256
Liver disease	186 (10.3%)	31 (5.2%)	33 (7%)	61 (16.2%)	61 (17%)	<0.001
Chronic obstructive pulmonary disease	25 (1.4%)	0 (0%)	4 (0.8%)	9 (2.4%)	12 (3.4%)	<0.001
Chronic kidney disease	20 (1.1%)	0 (0%)	0 (0%)	0 (0%)	20 (5.6%)	<0.001
Coinfections						
Mycobacteria	51 (2.8%)	22 (3.7%)	14 (3%)	6 (1.6%)	9 (2.5%)	<0.001
Pneumocystis	29 (1.6%)	7 (1.2%)	10 (2.1%)	8 (2.1%)	4 (1.1%)	0.804
Hepatitis B	151 (8.4%)	24 (4%)	54 (11.4%)	36 (9.6%)	37 (10.3%)	<0.001
Hepatitis C	421 (40.1%)	86 (14.5%)	185 (39.2%)	234 (62.2%)	216 (60.3%)	<0.001
Predisposing factors						
Congenital heart disease	8 (0.4%)	1 (0.2%)	1 (0.2%)	-	6 (1.7%)	0.008
Valve surgery	75 (4.3%)	21 (2.5%)	15 (3.2%)	16 (4.3%)	23 (6.4%)	0.029
Infection of cardiac device or implant	32 (1.8%)	4 (0.7%)	7 (1.5%)	8 (2.1%)	13 (3.6%)	<0.001
History of pacemaker or defibrillator placement	13 (0.7%)	2 (0.3%)	3 (0.6%)	1 (0.3%)	7 (2%)	0.025
History of prosthetic valve replacement	98 (5.8%)	25 (4.2%)	24 (5.1%)	19 (5.1%)	30 (8.4%)	0.009
Receiving intravenous therapy or home care	53 (3%)	7 (1.2%)	13 (2.8%)	16 (4.3%)	17 (4.7%)	<0.001
Hemodialysis dependent	31 (1.7%)	4 (0.7%)	5 (1.1%)	4 (1.1%)	18 (5%)	<0.001
Urgent admission	1593 (88.5%)	524 (88.2%)	429 (90.9%)	331 (88%)	309 (86.3%)	0.618
Surgical conditions	200 (11.1%)	65 (10.9%)	65 (13.8%)	42 (11.2%)	28 (7.8%)	0.162
Number of organ failures						
0	1528 (84.9%)	527 (88.7%)	409 (86.7%)	305 (81.1%)	287 (80.2%)	<0.001
1	228 (12.7%)	62 (10.4%)	53 (11.2%)	56 (14.9%)	57 (15.9%)	0.034
≥2	44 (2.9%)	5 (0.9%)	10 (2.4%)	15 (4.9%)	14 (4.9%)	0.003

Values are expressed as absolute count (percentage) and mean ± standard error mean. *p*-values: linear trend from 1997–1999 to 2008–2014 by the Extended Mantel–Haenszel Chi Square. Statistically significant differences are shown in bold.

## Supplementary Materials

The following are available online at https://www.mdpi.com/2077-0383/8/8/1167/s1, Table S1. Summary of ICD-9-CM coding used for baseline comorbidities investigated in this study, Table S2. Estimation of the number of people over 15 years of age infected with HIV in Spain, Table S3. Epidemiological trends of infective endocarditis in HIV-infected patients in Spain (1997 to 2014), Table S4. Epidemiological trends of causative microorganisms of infective endocarditis in HIV-infected patients in Spain (1997 to 2014), Table S5. Epidemiological trends of infective endocarditis (IE) in HIV-infected patients in Spain (1997 to 2014) stratified by modes of acquisition, Table S6. Epidemiological trends of causative microorganisms of infective endocarditis in HIV-infected patients in Spain (1997 to 2014) stratified by health care-associated and community-acquired endocarditis.

## References

[1] Holland T.L., Baddour L.M., Bayer A.S., Hoen B., Miro J.M., Fowler V.G. (2016). Infective endocarditis. Nat. Rev. Dis. Primers.

[2] Fernandez-Hidalgo N., Almirante B. (2012). Infective endocarditis in the xxi century: Epidemiological, therapeutic, and prognosis changes. Enferm. Infecc. Microbiol. Clin..

[3] Olmos C., Vilacosta I., Fernandez-Perez C., Bernal J.L., Ferrera C., Garcia-Arribas D., Perez-Garcia C.N., San Roman J.A., Maroto L., Macaya C. (2017). The evolving nature of infective endocarditis in spain: A population-based study (2003 to 2014). J. Am. Coll. Cardiol..

[4] Keller K., von Bardeleben R.S., Ostad M.A., Hobohm L., Munzel T., Konstantinides S., Lankeit M. (2017). Temporal trends in the prevalence of infective endocarditis in germany between 2005 and 2014. Am. J. Cardiol..

[5] Erichsen P., Gislason G.H., Bruun N.E. (2016). The increasing incidence of infective endocarditis in denmark, 1994-2011. Eur. J. Intern. Med..

[6] Dayer M.J., Jones S., Prendergast B., Baddour L.M., Lockhart P.B., Thornhill M.H. (2015). Incidence of infective endocarditis in england, 2000–2013: A secular trend, interrupted time-series analysis. Lancet.

[7] Fedeli U., Schievano E., Buonfrate D., Pellizzer G., Spolaore P. (2011). Increasing incidence and mortality of infective endocarditis: A population-based study through a record-linkage system. BMC Infect. Dis..

[8] Joffre J., Dumas G., Aegerter P., Dubee V., Bige N., Preda G., Baudel J.L., Maury E., Guidet B., Ait-Oufella H. (2019). Epidemiology of infective endocarditis in french intensive care units over the 1997–2014 period-from cub-rea network. Crit. Care.

[9] Deeks S.G., Overbaugh J., Phillips A., Buchbinder S. (2015). Hiv infection. Nat. Rev. Dis. Primers.

[10] Branas F., Azcoaga A., Garcia Ontiveros M., Antela A. (2018). Chronicity, ageing and multimorbidity. Enferm. Infecc. Microbiol. Clin..

[11] Bloch M. (2018). Frailty in people living with hiv. AIDS Res. Ther..

[12] Taramasso L., Tatarelli P., Di Biagio A. (2016). Bloodstream infections in hiv-infected patients. Virulence.

[13] Ribera E., Miro J.M., Cortes E., Cruceta A., Merce J., Marco F., Planes A., Pare J.C., Moreno A., Ocana I. (1998). Influence of human immunodeficiency virus 1 infection and degree of immunosuppression in the clinical characteristics and outcome of infective endocarditis in intravenous drug users. Arch. Intern. Med..

[14] Mocroft A., Youle M., Phillips A.N., Halai R., Easterbrook P., Johnson M.A., Gazzard B. (1998). The incidence of aids-defining illnesses in 4883 patients with human immunodeficiency virus infection. Royal free/chelsea and westminster hospitals collaborative group. Arch. Intern. Med..

[15] Mocroft A., Furrer H.J., Miro J.M., Reiss P., Mussini C., Kirk O., Abgrall S., Ayayi S., Bartmeyer B., Braun D. (2013). The incidence of aids-defining illnesses at a current cd4 count >/= 200 cells/mul in the post-combination antiretroviral therapy era. Clin. Infect. Dis..

[16] Gebo K.A., Burkey M.D., Lucas G.M., Moore R.D., Wilson L.E. (2006). Incidence of, risk factors for, clinical presentation, and 1-year outcomes of infective endocarditis in an urban hiv cohort. J. Acquir. Immune Defic. Syndr..

[17] Ministerio de Sanidad Consumo y Bienestar Social Sistema de Información de Atención Especializada (Siae). Https://www.Mscbs.Gob.Es/estadestudios/estadisticas/cmbdhome.Htm.

[18] Alvaro-Meca A., Palomares-Sancho I., Diaz A., Resino R., De Miguel A.G., Resino S. (2015). Pneumocystis pneumonia in hiv-positive patients in spain: Epidemiology and environmental risk factors. J. Int. AIDS Soc..

[19] Alvaro-Meca A., Berenguer J., Diaz A., Micheloud D., Aldamiz-Echevarria T., Fanciulli C., Resino S. (2017). Stroke in hiv-infected individuals with and without hcv coinfection in spain in the combination antiretroviral therapy era. PLoS ONE.

[20] Rockville (MD): Agency for Healthcare Research and Quality (US) Healthcare Cost and Utilization Project (hcup) Statistical Briefs. Https://www.Ncbi.Nlm.Nih.Gov/books/nbk367628/table/sb202.T4/.

[21] Díez M., Díaz A., Herrando I., Cornejo A. (2015). Hospital Survey of Patients with Hiv/Aids.

[22] Berenguer J., Alvarez-Pellicer J., Martin P.M., Lopez-Aldeguer J., Von-Wichmann M.A., Quereda C., Mallolas J., Sanz J., Tural C., Bellon J.M. (2009). Sustained virological response to interferon plus ribavirin reduces liver-related complications and mortality in patients coinfected with human immunodeficiency virus and hepatitis c virus. Hepatology.

[23] Berenguer J., Rodriguez-Castellano E., Carrero A., Von Wichmann M.A., Montero M., Galindo M.J., Mallolas J., Crespo M., Tellez M.J., Quereda C. (2017). Eradication of hepatitis c virus and non-liver-related non-acquired immune deficiency syndrome-related events in human immunodeficiency virus/hepatitis c virus coinfection. Hepatology.

[24] Thornton A.C., Jose S., Bhagani S., Chadwick D., Dunn D., Gilson R., Main J., Nelson M., Rodger A., Taylor C. (2017). Hepatitis b, hepatitis c, and mortality among hiv-positive individuals. AIDS.

[25] Nikolopoulos G.K., Paraskevis D., Hatzitheodorou E., Moschidis Z., Sypsa V., Zavitsanos X., Kalapothaki V., Hatzakis A. (2009). Impact of hepatitis b virus infection on the progression of aids and mortality in hiv-infected individuals: A cohort study and meta-analysis. Clin. Infect. Dis..

[26] Alvaro-Meca A., Rodriguez-Gijon L., Diaz A., Gil A., Resino S. (2014). Incidence and mortality of tuberculosis disease in spain between 1997 and 2010: Impact of human immunodeficiency virus (hiv) status. J. Infect..

[27] Diez M., Diaz A., Garriga C., Pons M., Ten A., Marcos H., Gutierrez G., Moreno S., Gonzalez-Garcia J., Barrios A. (2014). A low-cost, sustainable, second generation system for surveillance of people living with hiv in spain: 10-year trends in behavioural and clinical indicators, 2002 to 2011. Euro Surveill..

[28] El-Sadr W.M., Lundgren J., Neaton J.D., Gordin F., Abrams D., Arduino R.C., Babiker A., Burman W., Clumeck N., Cohen C.J. (2006). Cd4+ count-guided interruption of antiretroviral treatment. N. Engl. J. Med..

[29] Tumbarello M., Tacconelli E., Donati K.G., Citton R., Leone F., Spanu T., Cauda R. (2000). Hiv-associated bacteremia: How it has changed in the highly active antiretroviral therapy (haart) era. J. Acquir. Immune Defic. Syndr..

[30] Madeddu G., Monforte A.D., Girardi E., Di Biagio A., Lo Caputo S., Piolini R., Marchetti G., Pellizzer G., Giacometti A., Galli L. (2014). Cd4 cell count and the risk of infective and non-infective serious non-aids events in hiv-positive persons seen for care in italy. J. Int. AIDS Soc..

[31] Kanters S., Vitoria M., Doherty M., Socias M.E., Ford N., Forrest J.I., Popoff E., Bansback N., Nsanzimana S., Thorlund K. (2016). Comparative efficacy and safety of first-line antiretroviral therapy for the treatment of hiv infection: A systematic review and network meta-analysis. Lancet HIV.

[32] Iacob S.A., Iacob D.G., Jugulete G. (2017). Improving the adherence to antiretroviral therapy, a difficult but essential task for a successful hiv treatment-clinical points of view and practical considerations. Front. Pharmacol..

[33] Tsabedze N., Vachiat A., Zachariah D., Manga P. (2018). A new face of cardiac emergencies: Human immunodeficiency virus-related cardiac disease. Cardiol. Clin..

[34] Vogkou C.T., Vlachogiannis N.I., Palaiodimos L., Kousoulis A.A. (2016). The causative agents in infective endocarditis: A systematic review comprising 33,214 cases. Eur. J. Clin. Microbiol. Infect. Dis..

