# Supplementary material for: National Temporal Trend Analysis of Infective Endocarditis among Patients Infected with HIV in Spain (1997–2014): A Retrospective Study
Source: J Clin Med. 2019 Aug 4;8(8):1167. doi: 10.3390/jcm8081167 (PMC6723534; doi:10.3390/jcm8081167)
Supplement: Supplementary file 1 [file jcm-08-01167-s001.pdf]

Supplementary Table 1. Summary of ICD-9-CM coding used for baseline comorbidities investigated in this study.

Description	Diagnosis codes (index or prior admissions)	Procedure codes (prior admissions)
**HIV infection**	042 or V08	
**Endocarditis**	421.0, 421.1, 421.9	
**Abuse of alcohol and drugs**		
Abuse of drugs	292.x, 304.x, 305.x, and 965.0x	
Abuse of alcohol	305.0,303.0,303.9,291.0,291.1,291.2,291.3,291.4,291.5,291.8,291.9,571.0,571.1,571.2,571.3,425.5,535.3,357.5,265.2, V11.3,790.3,980.0	
Abuse of tobacco	305.1, V15.82	
**Conditions influencing in health status**		
Surgical conditions	V42, V45	
Trauma	E880* to E929*, E950 to E999*	
**Comorbidities**		
Diabetes	250.x	
Hypertension	401.x, 402.x, 403.x, 404.x, 405.x, and 437.2	
Coronary artery disease	410.x to 414.x, 429.5, 429.6, 429.7, 429.71, 429.79, V45.81 and V45.82	
Peripheral vascular disease	093.0, 437.3, 440.x, 441.x, 443.1, 443.2, 443.8, 443.9, 447.1, 557.1, 557.9, 996.1, 996.62, 996.74, and V43.4	
Cerebrovascular Disease	362.34, 430, 431, 432, 433, 434, 435, 436, 437, 438	
Cancer	140.x to 209.x	
Liver disease	070.x, 456.x, 571.x, 572.x, 573.x, and V42.7	
Chronic obstructive pulmonary disease	491x, 492.x, 493.x, 494.x and 496.x	
Chronic kidney disease	403.x, 404.x, 585.x, V42.0, V56.x, and V45.1x	
**Predisposing factor**		
Congenital Heart Disease	745.10, 745.11, 745.12, 745.19, 745.2, 745.3, 745.4, 745.5, 745.6, 745.60, 745.61, 745.69, 745.7, 745.8, 745.9, 746.00, 746.01, 746.02, 746.09, 746.1, 746.2, 746.3, 746.4, 746.5, 746.6, 746.7, 746.8, 746.81, 746.82, 746.83, 746.84, 746.85, 746.86, 746.87, 746.89, 746.9, 747.0, 747.1, 747.10, 747.11, 747.2, 747.20, 747.21, 747.22, 747.29, 747.3, 747.31, 747.32, 747.39, 747.4, 747.40, 747.41, 747.42, 747.49, 747.5, 747.9, V13.65	35.4, 35.41, 35.42, 35.5, 35.50, 35.51, 35.52, 35.53, 35.54, 35.55, 35.6, 35.60, 35.61, 35.62, 35.63, 35.7, 35.70, 35.71, 35.72, 35.73, 35.81, 35.82, 35.83, 35.84, 35.9, 35.91, 35.92, 35.93, 35.94, 35.95, 35.98, 35.99, 39.0 and 39.21

Valve Surgery		35.2, 35.20, 35.21, 35.22, 35.23, 35.24, 35.25, 35.26, 35.27, 35.28, 35.14, 35.13, 35.10, 35.11, 35.12, 35.31, 35.32 and 35.33
Complications due to cardiac device	996.72	
Infection of cardiac device or implant	996.61	
History of pacemaker or defibrillator placement	V45.01, V53.31, V45.02, V53.32, 996.01 and 996.04	37.70, 37.71, 37.72, 37.73, 37.74, 37.75, 37.76, 37.80, 37.81, 37.82, 37.83, 37.85, 37.86, 37.87, 37.94, 37.95, 37.96, 37.97, 37.98, 00.50, 00.51, 00.53 and 00.54
History of prosthetic valve replacement	V43.2, V43.21, V43.22, V43.3, 996.02, 996.71	35.2, 35.20, 35.21, 35.22, 35.23, 35.24, 35.25, 35.26, 35.27, and 35.28
Receiving intravenous therapy or home care	V55, V55.0, V55.1, V55.2, V55.3, V55.4,V55.5, V55.6, V55.7, V55.8 and V55.9, V56.1, V58.62, V58.11, V58.12, V58.81, V66.2, V67.2, 996.73, 996.74, 996.62, 999.3, 999.31, 999.32, 999.33, 999.39	00.15, 17.70 and 99.25
Hemodialysis dependent	585.6, V45.1, V45.11, V45.12, V56.0, V56.1 and V56.31	39.95
**Organ failure**		
Cardiovascular	427.5, 458.0, 458.8, 458.9, 785.5, 796.3	
Hematologic	286.2, 286.6, 286.9, 287.3, 287.4, 287.5, 790.92	
Hepatic	570, 572.2, 573.3, 573.4	
Neurologic	293, 348.1, 348.3, 780.01, 780.09, 89.14	
Renal	580, 580.0, 580.4, 580.8, 580.81, 580.89, 580.9, 584, 584.5, 584.6, 584.7, 584.8, 584.9, 586, 39.95	
Respiratory	518.5, 518.8, 786.03, 799.1, 786.09, 96.7, 96.71, 96.72, 96.04, 93.90	
Metabolic	276.2	
**Microorganisms that cause endocarditis**		
Staphylococcus	038.1, 038.10, 038.11, 038.12, 038.19, 041.1, 041.11, 041.12, 041.10 and 041.19	
Staphylococcus aureus	038.11, 038.12, 041.11, and 041.12	
Streptococcus	038.0, 038.2, 041.0, 041.00, 041.01, 041.02, 041.03, 041.05, 041.09 and 041.2	
Enterococcus	041.04	
Gram negative bacilli	038.4, 038.40, 038.41, 038.42, 038.43, 038.44, 038.49, 041.3, 041.4, 041.5, 041.6, 041.7 and 041.85	
Fungus	112.5, 112.81, 116.0, 115.04, 115.14, 115.94 and 117.3	

**Other coinfections not related to infective endocarditis**	
**Mycobacteria**	
Tuberculosis	011.x-018.x
Diseases due to other mycobacteria	031.x
**Herpes infections**	
Herpes zoster	053.x
Herpes simplex	054.x
Cytomegaloviral disease	078.5
**Protozoa**	
Leishmaniasis	085.0
Toxoplasmosis	130.x
**Fungus**	
Pneumocystosis	136.3
Candidiasis of mouth	112.0
Candidiasis of other sites	112.x (Except 112.0, 112.5, and 112.81)
Other mycoses	117.x
**Viral hepatitis**	
Hepatitis B	070.2x, 070.3x, or V02.61
Acute hepatitis C	070.51
Chronic hepatitis C	070.44, 070.54, 070.7x, or V02.62

Supplementary Table 2. Estimation of the number of people over 15 years of age infected with HIV in Spain.

Year	HIV-infected patients (No.) (*)
**1997**	88,158
**1998**	90,652
**1999**	93,359
**2000**	96,670
**2001**	100,404
**2002**	104,447
**2003**	108,375
**2004**	112,415
**2005**	116,396
**2006**	120,457
**2007**	124,461
**2008**	128,240
**2009**	131,546
**2010**	133,968
**2011**	135,968
**2012**	137,640
**2013**	139,318
**2014**	141,290

(*), The estimation of the number of people living with HIV/AIDS in Spain was provided by the National Centre of Epidemiology (Instituto de Salud Carlos III, Madrid, Spain). This estimation was done using the Estimation and Projection Package (EPP) and Spectrum software, two programs developed by the Joint UNAIDS/WHO for estimating and projecting HIV prevalence at country level [1,2].

BIBLIOGRAPHY

1. Brown, T.; Bao, L.; Raftery, A.E.; Salomon, J.A.; Baggaley, R.F.; Stover, J.; Gerland, P. Modelling hiv epidemics in the antiretroviral era: The unaids estimation and projection package 2009. *Sex. Transm. Infect.* **2010**, *86 Suppl 2*, ii3-10.
2. Stover, J. Projecting the demographic consequences of adult hiv prevalence trends: The spectrum projection package. *Sex. Transm. Infect.* **2004**, *80 Suppl 1*, i14-18.

Supplementary Table 3. Epidemiological trends of infective endocarditis in HIV-infected patients in Spain (1997 to 2014).

	**Hospital admissions** (per 10,000 patients/year)		**Incidence** (per 10,000 patients/year)		**Mortality** (per 100,000 patients/year)		**Case fatality rate** (%)	
	**No.**	**Rate (95%CI)**	**No.**	**Rate (95%CI)**	**No.**	**Rate (95%CI)**	**No.**	**Rate (95%CI)**
**Whole follow-up**	1800	8.5 (8.2;8.9)	1439	6.8 (6.5; 7.2)	232	11 (9.6; 12.4)	232	12.9 (11.5; 14.6)
1997-1999	594	21.8 (20.1; 23.6)	496	18.2 (16.7; 19.9)	65	23.9 (18.6; 30.2)	65	10.9 (8.6; 13.6)
2000-2003	472	11.5 (10.5; 12.6)	371	9.1 (8.2; 10)	64	15.6 (12.1; 19.8)	64	13.5 (10.6; 16.8)
2004-2007	376	7.9 (7.2; 8.7)	295	6.2 (5.5; 6.9)	51	10.8 (8.1; 14)	51	13.6 (10.4; 17.3)
2008-2014	358	3.8 (3.4; 4.2)	277	2.9 (2.6; 3.3)	52	5.5 (4.1; 7.1)	52	14.5 (11.2; 18.5)
**P-values** (*)								
97-99 vs. 00-03		**<0.001**		**<0.001**		0.019		0.193
97-99 vs. 04-07		**<0.001**		**<0.001**		**<0.001**		0.220
97-99 vs. 08-14		**<0.001**		**<0.001**		**<0.001**		0.103
00-03 vs. 04-07		**<0.001**		**<0.001**		0.057		0.998
00-03 vs. 08-14		**<0.001**		**<0.001**		**<0.001**		0.691
04-07 vs. 08-14		**0.006**		**<0.001**		**<0.001**		0.707
**P-values** (§)								
Linear trend		**<0.001**		**<0.001**		**<0.001**		0.112

Values are expressed as absolute count and rate (95% confidence interval (95% CI)).
P-values: (*), differences by the Chi Square test; (§), linear trend from 1997-1999 to 2008-2014 by the Extended Mantel Haenszel Chi Square.
Statistically significant differences are shown in bold.

Supplementary Table 4. Epidemiological trends of causative microorganisms of infective endocarditis in HIV-infected patients in Spain (1997 to 2014).

	Staphylococcus aureus (%)		**Coagulase-negative staphylococci** (%)		**Streptococci** (%)		**Gram-negative bacilli** (%)		**Enterococci** (%)		**Fungus** (%)	
	**No.**	**Rate (95%CI)**	**No.**	**Rate (95%CI)**	**No.**	**Rate (95%CI)**	**No.**	**Rate (95%CI)**	**No.**	**Rate (95%CI)**	**No.**	**Rate (95%CI)**
**Whole follow-up**	768	42.7 (40.4; 45.0)	132	7.3 (6.2; 8.6)	167	9.3 (8.2; 10.5)	146	8.3 (7.1;9.6)	54	3.0 (2.4; 3.7)	26	1.4 (1.0 ;2.1)
1997-1999	233	39.2 (35.4; 43.2)	73	12.3 (9.8; 15.1)	45	9.2 (6.9; 12.0)	37	6.2 (4.5; 8.4)	11	2.2 (1.2; 3.9)	7	0.5 (0.1; 1.3)
2000-2003	201	42.6 (38.2; 47.1)	25	5.3 (3.5; 7.6)	36	9.4 (6.8; 12.6)	33	7 (5; 9.6)	10	2.6 (1.4; 4.6)	2	1.5 (0.7; 2.9)
2004-2007	184	48.9 (43.9; 54.0)	19	5.1 (3.2; 7.6)	38	12.7 (9.3; 16.8)	30	8 (5.6; 11)	11	3.7 (2.0; 6.3)	14	0.5 (0.1; 1.7)
2008-2014	150	41.9 (36.9; 47.1)	15	4.2 (2.5; 6.6)	48	16.3 (12.4; 20.9)	49	13.7 (10.4; 17.5)	22	7.5 (4.9; 10.9)	3	3.9 (2.3; 6.3)
**P-values** (*)												
97-99 vs. 00-03		0.267		**<0.001**		0.913		0.618		0.726		0.118
97-99 vs. 04-07		**0.003**		**<0.001**		0.118		0.295		0.235		0.999
97-99 vs. 08-14		0.415		**<0.001**		**0.003**		**<0.001**		**<0.001**		**<0.001**
00-03 vs. 04-07		0.065		0.874		0.167		0.586		0.423		0.312
00-03 vs. 08-14		0.843		0.461		**0.007**		**0.001**		**0.003**		**0.027**
04-07 vs. 08-14		0.056		0.578		0.211		**0.013**		**0.043**		**0.002**
**P-values** (§)												
Linear trend		0.106		**<0.001**		**0.001**		**<0.001**		**0.003**		**<0.001**

Values are expressed as absolute count and rate (95% confidence interval (95% CI)).
P-values: (*), differences by the Chi Square test; (§), linear trend from 1997-1999 to 2008-2014 by the Extended Mantel Haenszel Chi Square.
Statistically significant differences are shown in bold.

Supplementary Table 5. Epidemiological trends of infective endocarditis (IE) in HIV-infected patients in Spain (1997 to 2014) stratified by modes of acquisition.

	**Community-acquired IE** (%)		**Healthcare-associated IE** (%)		**p-value**
	**No.**	**Rate (95%CI)**	No.	Rate (95%CI)	**No.**
**Whole follow-up**	823	45.7 (43.4; 48.0)	823	45.7 (43.4; 48.0)	**<0.001**
1997-1999	276	46.5 (42.5; 50.5)	318	53.5 (50.2; 56.9)	**0.016**
2000-2003	264	55.9 (51.4; 60.4)	208	44.1 (40.4; 47.8)	**<0.001**
2004-2007	253	67.3 (62.4; 71.9)	123	32.7 (28.8; 36.8)	**<0.001**
2008-2014	184	51.4 (46.4; 56.5)	174	48.6 (44.3 ; 52.9)	0.454
**P-values** (*)					
97-99 vs. 00-03		**0.002**		0.073	
97-99 vs. 04-07		**<0.001**		**<0.001**	
97-99 vs. 08-14		0.140		0.403	
00-03 vs. 04-07		**0.001**		**0.025**	
00-03 vs. 08-14		0.194		0.431	
04-07 vs. 08-14		**<0.001**		**0.004**	
**P-values** (§)					
Linear trend		**0.001**		**<0.001**	

Values are expressed as absolute count and rate (95% confidence interval (95% CI)).
P-values: (*), differences by the Chi Square test; (§), linear trend from 1997-1999 to 2008-2014 by the Extended Mantel Haenszel Chi Square.
Statistically significant differences are shown in bold.

Supplementary Table 6. Epidemiological trends of causative microorganisms of infective endocarditis in HIV-infected patients in Spain (1997 to 2014) stratified by healthcare-associated and community-acquired endocarditis.

	Staphylococcus aureus (%)		**Coagulase-negative staphylococci** (%)		**Streptococci** (%)		**Gram-negative bacilli** (%)		**Enterococci** (%)		**Fungus** (%)	
	**No.**	**Rate (95%CI)**	**No.**	**Rate (95%CI)**	**No.**	**Rate (95%CI)**	**No.**	**Rate (95%CI)**	**No.**	**Rate (95%CI)**	**No.**	**Rate (95%CI)**
A) <u>Healthcare-associated</u>												
**Whole follow-up**	284	34,5 (31.3; 37.8)	74	9,0 (7.2; 11.1)	71	8.6 (7.1; 10.4)	62	7,5 (5.9; 9.5)	20	2.4 (1.7; 3.5)	16	1,9 (1.2; 3.1)
1997-1999	101	31.8 (26.8; 37)	45	14.2 (10.7; 18.3)	25	9.6 (6.5; 13.6)	19	6 (3.8; 9)	6	2.3 (1.0; 4.7)	2	0.6 (0.1; 2)
2000-2003	67	32.2 (26.1; 38.8)	11	5.3 (2.8; 9)	13	8.3 (4.8; 13.4)	18	8.7 (5.4; 13)	3	1.9 (0.5; 5.0)	7	3.4 (1.5; 6.5)
2004-2007	49	39.8 (31.5; 48.6)	7	5.7 (2.6; 10.8)	11	12.4 (6.7; 20.4)	7	5.7 (2.6; 10.8)	3	3.4 (1.0; 8.7)	1	0.8 (0.1; 3.7)
2008-2014	67	38.5 (31.5; 45.9)	11	6.3 (3.4; 10.7)	22	15.6 (10.3; 22.3)	18	10.3 (6.5; 15.5)	8	5.7 (2.7; 10.4)	6	3.4 (1.5; 7)
p-values (§)		0.069		**0.001**		0.055		0.173		0.064		**0.048**
B) <u>Community-acquired</u>												
**Whole follow-up**	484	49.5 (46.4; 52.7)	58	5.9 (4.6; 7.6)	96	9.6 (8.3; 11.5)	87	8.9 (7.2; 10.8)	34	3.4 (2.6; 4.6)	10	1.0 (0.5; 1.8)
1997-1999	132	47.8 (42; 53.7)	28	10.1 (7; 14.1)	20	8.7 (5.6; 12.9)	18	6.5 (4.1; 9.9)	5	2.2 (0.8; 4.7)	1	0.4 (0; 1.7)
2000-2003	134	50.8 (44.7; 56.8)	14	5.3 (3.1; 8.5)	23	10.1 (6.7; 14.6)	15	5.7 (3.4; 9)	7	3.1 (1.4; 6.0)	0	0 (0; 0)
2004-2007	135	53.4 (47.2; 59.4)	12	4.7 (2.6; 7.9)	27	12.9 (8.8; 17.9)	23	9.1 (6; 13.1)	8	3.8 (1.8; 7.1)	1	0.4 (0; 1.8)
2008-2014	83	45.1 (38; 52.3)	4	2.2 (0.7; 5.1)	26	17.0 (11.7; 23.5)	31	16.8 (12; 22.8)	14	9.2 (5.3; 14.5)	8	4.3 (2.1; 8)
p-values (§)		0.886		**<0.001**		**0.010**		**<0.001**		**0.002**		**<0.001**
p-values (*)												
**Whole follow-up**		**<0.001**		**0.013**		0.654		0.293		0.285		0.103
1997-1999		**<0.001**		0.138		0.747		0.783		0.931		0.648
2000-2003		**<0.001**		0.994		0.553		0.209		0.484		**0.003**
2004-2007		**0.014**		0.694		0.906		0.254		0.999		0.601
2008-2014		0.206		**0.050**		0.747		0.074		0.277		0.661

Values are expressed as absolute count; and rate (95% confidence interval (95% CI)).
P-values: (*), differences between groups were calculated by the Chi Square test. (§), Linear trends from 1997-1999 to 2008-2014 calculated by the Extended Mantel Haenszel Chi Square. Statistically significant differences are shown in bold.

This supplementary material is hosted by Eurosurveillance as supporting information alongside the article [**National temporal trend analysis of infective endocarditis among patients infected with HIV in Spain (1997-2014): a retrospective study**] on behalf of the authors who remain responsible for the accuracy and appropriateness of the content. The same standards for ethics, copyright, attributions and permissions as for the article apply. Eurosurveillance is not responsible for the maintenance of any links or email addresses provided therein.
